# Phytochemical analysis and antimicrobial activity of *Silybum marianum* L. via multi-solvent extraction

**DOI:** 10.1186/s13568-025-01925-2

**Published:** 2025-08-20

**Authors:** Nashaat N. Mahmoud, Mohamed T. Selim

**Affiliations:** Botany and Microbiology Department, Faculty of Science, Al-Azhar University, Nasr City, Cairo 11884 Egypt

**Keywords:** *S. marianum* L., Phytochemical, Carbohydrates, Lipids, Proteins, Antimicrobial

## Abstract

Milk thistle (*Silybum marianum* L.) is an essential medicinal plant belonging to the family Asteraceae. The active ingredient of milk thistle is silymarin, a key component used to treat numerous physical and biological ailments. This study aimed to compare the nutritional composition, total phenolic, flavonoid, and tannin contents, and the antimicrobial activities of *S. marianum* stems, leaves, and flowers extracted using five different solvents. Phytochemical assays were used to evaluate the total phenolic, flavonoid, and tannin contents in petroleum ether, ethyl acetate, acetone, methanol, and water extracts from different plant parts. The proximate composition showed that the leaves had a higher percentage of moisture (11.53%). In contrast, the flowers showed increased lipid content, carbohydrate, and protein concentration (5.17, 69.86, and 10.03%, respectively), and stems showed elevated ash content (28.67%) compared to leaves and flowers. In *S. marianum*, the highest yield was obtained from the flowers, while the leaves and stems produced progressively lower amounts, respectively. Among the solvents tested, extraction with water made the largest yield, followed in decreasing order by methanol, acetone, ethyl acetate, and petroleum ether. The findings of the study revealed that alkaloids, tannins, flavonoids, glycosides, steroids, quinones, phenols, anthraquinones, cardiac glycosides, and terpenoids were found by phytochemical analysis of *S. marianum* in different parts. At the same time, saponins and anthocyanins were completely absent in all parts. On the other hand, coumarins are present in leaves and flowers and are completely lacking in stems. The highest levels of phenol content, tannins, and flavonoids were found in the methanol extract of the flowers (183.12 ± 11.02 mg gallic acid equivalent/g (mg GAE/g), 187.43 ± 15.91 mg quercetin equivalents/g (mg QE/g), and 94.40 ± 16.04 mg TAE/g, respectively). In contrast, the water extract of stems had the lowest amount (5.45 ± 1.32 mg GAE/g, 9.60 ± 1.5 mg QE/g, and 3.27 ± 1.53 mg TAE/g, respectively). Antimicrobial tests revealed the extract's ability to inhibit several Gram-positive bacteria (*S. aureus* ATCC 6538 and *B.subtilis* ATCC 6633), Gram-negative bacteria (*P. aeruginosa* ATCC 9027, *S. typhimurium* ATCC 14028, *E. coli* ATCC 11229), and eukaryotic strains such as unicellular fungi (*C.albicans* ATCC 10231). These results confirm the potential of milk thistle extract as a naturally occurring antimicrobial agent.

**Graphical abstract:**

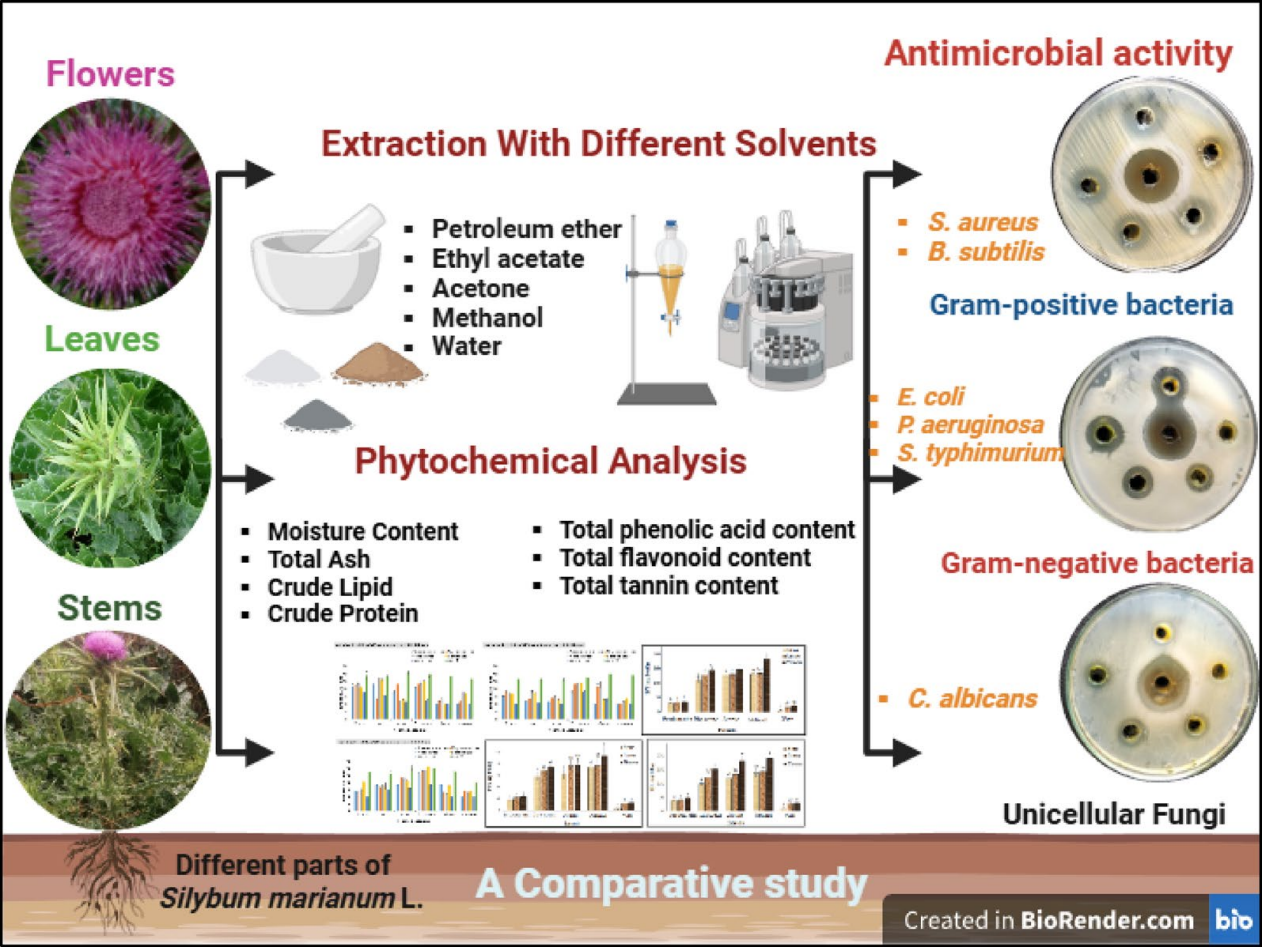

**Supplementary Information:**

The online version contains supplementary material available at 10.1186/s13568-025-01925-2.

## Introduction

Many extracts from medicinal plants contain primary active compounds or secondary metabolites that can help fight infectious diseases (Elkady et al. 2024). Due to their rich content of various phytochemicals, herbs, and shrubs serve as an important resource for treating multiple diseases (Sharaf et al. 2022). *S marianum* L. is a significant and historic medicinal plant among these species. *S marianum* (Asteraceae family) is a Mediterranean native plant used extensively for its hepatoprotective properties. Silymarin, the main bioactive component of milk thistle, is a complex blend of flavonolignans known for its neuroprotective, anti-inflammatory, and antioxidant properties (Lucia and Rita 2021). This plant will tolerate harsh conditions like salinity, drought, and cold (Martinelli 2019; Papademos and Golia 2024). Many ailments have long been treated with milk thistle. Several studies suggest that milk thistle may aid in liver regeneration and protection (Das and Mukherjee 2012; Kwon et al. 2013).

Silybin, isosilybin, and silychristin are all members of the silymarin class of flavonolignans. Dihydroflavonol derivatives undergo dehydration condensation to produce these compounds (Biedermann et al. 2014). Hepatoprotective, anti-inflammatory, and antioxidant properties are all displayed by silymarin (Karimzadeh et al. 2024; Ma et al. 2024). For thousands of years, *S. marianum* has been utilized as a medicinal herb; it was initially used to treat venomous snake bites and protect the liver (Morazzoni et al. 1995). The way silymarin works has become more apparent with the advancement of modern medicine. When taken as a liver protectant, silymarin can protect the liver cell membrane, prevent liver cell fibrosis, and promote liver cell regeneration or repair (de Avelar et al. 2023).

In early research, silymarin has demonstrated potential as an antibacterial agent against gram-negative and gram-positive bacteria (Iraqi et al. 2025). When paired with ampicillin or oxacillin, silybin exhibited increased synergistic action against Methicillin-resistant *S. aureus* (MRSA) (Kang et al. 2011). Silibinin decreased the Gram-positive bacteria's production of macromolecules, such as proteins and RNA (Lee and Liu 2003).

This investigation sought to assess the in vitro proximate compositions, phytochemical analysis, and antimicrobial potential of the dried stems, leaves, and flowers of *S. marianum* extracted with different solvents. To our knowledge, this is the first comprehensive comparative study investigating the phytochemical and antimicrobial profiles of three different parts (stems, leaves, and flowers) of *S. marianum* using five different solvents with varying polarities. Our study offers several unique contributions. (1) The comparative analysis of underexplored plant parts (stems, leaves, and flowers) of *S. marianum* under the same experimental conditions. (2) A wide range of solvents with different polarities is used to evaluate extraction efficiency. (3) The broad-spectrum antimicrobial activity assessment, including bacteria and fungi. (4) The correlation between phytochemical content and biological activity. (5) These aspects collectively represent a novel contribution not previously addressed in a single study. So, this dual-layer Comparison (solvent and plant part) contributes a new dimension to the field and offers practical guidance for targeted extraction and pharmaceutical applications.

## Materials and methods

### Plant material

The air-dried *S. marianum* L. stems, leaves, and flowers (Family: Asteraceae) were collected from 59 km, Alexandria-Cairo Desert Road, Governorate, Egypt. The formal identification of *S. marianum* has been conducted by Dr. Nashaat Nasrallah Mahmoud, Lecturer of Botany, Faculty of Science, Botany and Microbiology Department, Cairo, Egypt. A voucher herbarium specimen (AZU/SCI/BOT/HERB/2024-027) was archived in the Herbarium at the Department of Botany and Microbiology, Faculty of Science, Al-Azhar University, Cairo, Egypt. The fresh aerial flowering parts (stems, leaves, and flowers) of *S. marianum* were investigated during the investigation period in March 2024, after being cleaned with distilled water (DW(, the components were dried at lab temperature and shaded until their weight remained constant. An electric blender was used to crush the dried ingredients into a fine powder, which was then sieved and Maintained at room temperature in a dry glass jar for later use.

## Methods

### Preparation of extracts

**Fig. 1:**
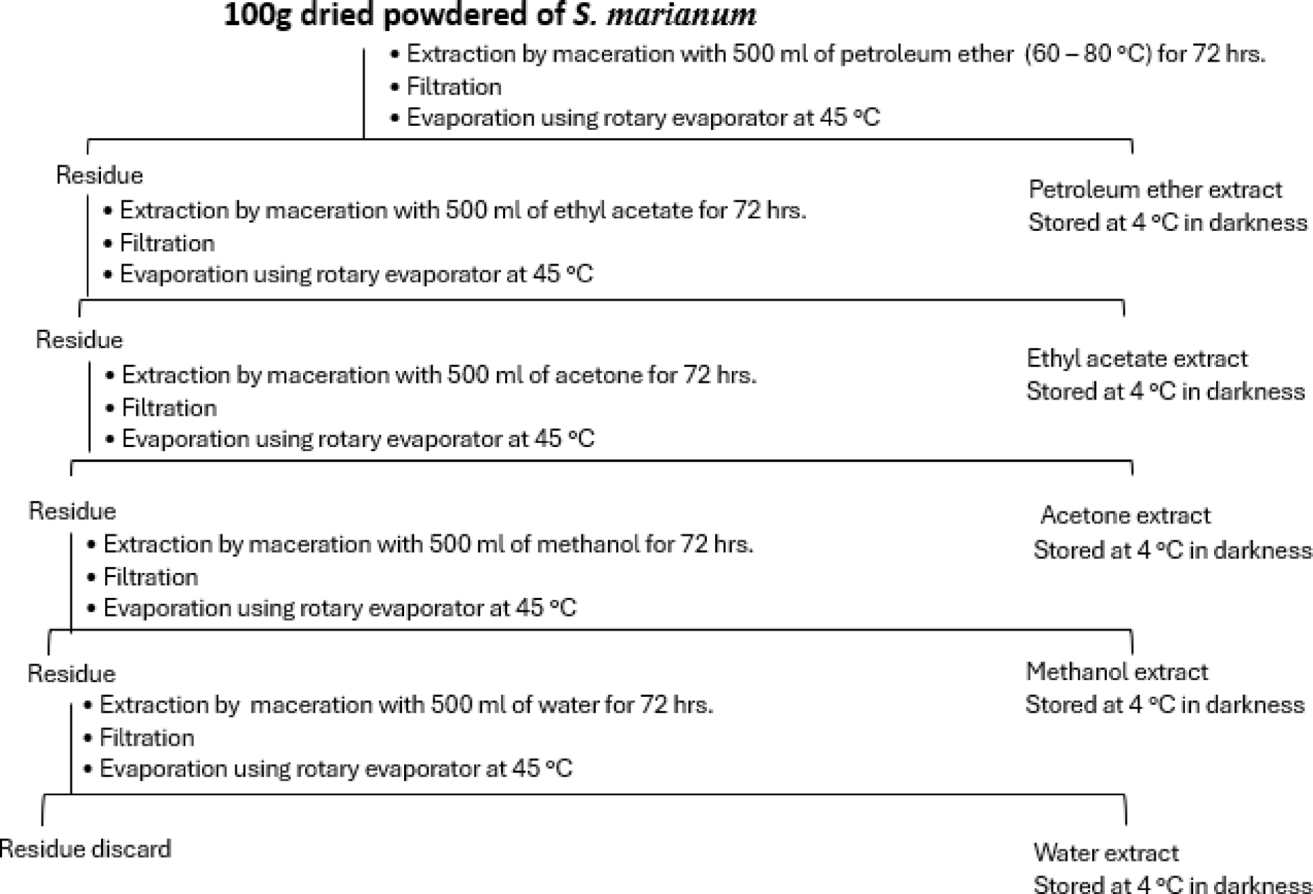
Schematic diagram of the successive extraction of dried powdered *S. marianum*

The powders of stems, leaves, and flowers (100 g each), from *S. marianum* were each extracted at room temperature by a maceration method with 500 mL petroleum ether (60–80 ℃) for 72 h with occasional shaking. After passing the extract through the Whatman No. 1 filter paper, the residue was extracted again using the same procedure and additional solvents, namely ethyl acetate, acetone, methanol, and water, until all plant materials had been used up (Fig. 1). The filtrate was collected using a rotary evaporator, and pressure and temperature of no more than 45 °C were used to remove the excess solvent. The yield was then calculated using the following formula (Eq. 1). The collected samples were kept in a dark, 4 °C environment to examine their antimicrobial and phytochemical properties (Pizzale et al. 2002).

1 $$ $$$$ \begin{aligned} & \% {\text{ Yield }} \\ & \quad {\text{ = [}}\left( {{\text{Weight of the extract obtained}}} \right)\\ &\qquad /\left( {{\text{Weight of the sample taken}}} \right){\text{]}} \\ & \quad \times 100 \\ \end{aligned} $$

## Nutritional assays

### Moisture content

Moisture content was measured by using the method described by Arunachalam and Parimelazhagan (2014), with minor modifications. Quickly record 10 g of the fresh sample as the "fresh weight of the sample." The sample must be dried using a suitable drying apparatus to a consistent weight at a maximum temperature of 115 °C. Allow the sample time to cool. Discover the "dry weight of the sample" by weighing the cooled sample again. Using the following formula (Eq. 2), the sample's moisture content is determined:

2 $$\begin{aligned} &\% {\text{ Moisture }}\\ & \quad = [({\text{Fresh weight of sample }} \\ & \qquad- {\text{Dry weight of sample}})\\ &\qquad/\left( {{\text{Fresh weight of sample}}} \right)] \\ & \quad \times 100 \\ \end{aligned} $$

### Total ash

Weighed 5 g of dried powder and were burnt to 600 °C in a furnace for three hours, and then the mixture was cooled. Concerning the air-dried sample, the ash was weighed, and the percentage was measured (Eq. 3) (Premnath and Gomez 2012).

3 $$ \begin{aligned} & \% {\text{ Ash }} \\ & \quad = [({\text{Sample weight after burning}})\\ & \qquad /({\text{Sample weight before burning}}) \\ & \quad \times 100 \\ \end{aligned} $$

### Crude lipid

The crude lipid content of various parts of *S. marianum* was estimated by Arunachalam and Parimelazhagan (2014) with slight modifications. 10 g of dry sample was extracted for 6 h using 250 mL of petroleum ether (60–80 °C). Following the extraction, the flask contents were dried in a 102 °C oven until the weight remained constant, allowing the solvent to be removed. The flask was then allowed to cool in a desiccator. After cooling, the flask and its contents were weighed. The crude lipid content was calculated using the formula below (Eq. 4):

4 $$ \begin{aligned}& \% {\text{ Crude lipid}} \\ & \quad = [({\text{Weight of flask and extracted lipid }} \\ & \qquad - {\text{ Weight of empty flask}})\\ & \qquad/({\text{Weight of sample taken}})] \\ & \quad \times 100 \\ \end{aligned} $$

### Crude protein

The amount of crude protein in various *S. marianum* parts was determined by Krishna et al. (2014). Using a mortar and pestle, 10 mL of 0.2 M phosphate buffer was used to grind 100 mg of the sample powder. After filtering the mixture, the filtrate was centrifuged for ten minutes at 3000 × g. After collecting the supernatant, DW was added to bring the volume down to 10 mL. Then, about 1 ml of the upper liquid was diluted to 10 ml with DW and used for protein estimation. A working standard solution of bovine serum albumin (BSA) (200 μg/mL) was prepared in distilled water. 1 mL of the protein extract and standard solutions (0–200 μg/mL) were placed into separate test tubes for the protein assay. Each test tube, including the blank, was filled with 5 mL of alkaline copper solution. Each test tube was thoroughly vortexed and incubated the mixture for 10 min. Each tube was filled with 0.5 mL of Folin-Ciocalteu reagent (1 N). The tubes were thoroughly mixed, and then they were left to incubate for 30 min at room temperature in the dark. A spectrophotometer was used to measure the absorbance of the blue colour that developed at 660 nm. The carbohydrate content is calculated by difference: 100 − (moisture content + crude protein + crude fat + total ash). The method described by Indrayan et al. (2005), was used to assess the samples' nutritional value.

## Secondary metabolite quantifications

### Preliminary phytochemical screening

The defined protocol was used to perform a preliminary screening for the presence of alkaloids, flavonoids, phytosterols, saponins, phenolic compounds, tannins, terpenoids, carbohydrates, oils, and lipids (Abdelaziz et al. 2023; Mohamed et al. 2023; Trease and Evans 1989).

#### Flavonoids

The lead acetate test was performed by adding 1 mL of 10% lead acetate solution to 3 mL of alcoholic extract. A yellow or white precipitate indicated the presence of flavonoids. For the NaOH test, 2 mL of aqueous NaOH, followed by diluted HCl, was added to 2 mL of the extract. A yellow-orange colouration that turned colorless upon the addition of diluted HCl indicated the presence of flavonoids. Additionally, Shinado's test was conducted by adding a few drops of concentrated hydrochloric acid and small magnesium turnings to the alcoholic extract, followed by boiling for five minutes. A red coloration confirmed the presence of flavonoids.

#### Saponins

In the froth test, 2.5 g of dried plant powder was boiled in 20 mL of distilled water using a water bath and then filtered. After cooling, a portion of the extract was vigorously shaken in a test tube. The formation of stable foam indicated the presence of saponins.

#### Tannins

Ferric chloride test was conducted by adding three to four drops of ferric chloride solution to the extract. A green or bluish-black color indicated the presence of tannins.

#### Phenols

The Folin–Ciocalteu test was performed by mixing 1 mL of extract, Folin–Ciocalteu reagent, and 20% Na_2_CO_3_ in a clean test tube. The development of a dark blue color confirmed the presence of phenolic compounds.

#### Glycosides

Glycosides were tested by adding 1 mL of water to a small amount of the extract, followed by thorough shaking. Aqueous sodium hydroxide was then added, and the appearance of a yellow coloration indicated the presence of glycosides. In Borntrager's test for glycosides, 3 mL of aqueous extract was treated with diluted sulfuric acid, boiled, and filtered. An equal volume of benzene was added to the filtrate and shaken thoroughly. The organic layer was separated and treated with an equal amount of diluted ammonia. A pink coloration in the ammonia layer indicated the presence of glycosides.

For Cardiac Glycosides, three mL of alcoholic extract was diluted with distilled water, and 0.5 mL of concentrated lead acetate was added to remove chlorophyll and pigments. Excess lead was removed by adding 10% H_2_SO_4_ dropwise until no further precipitation occurred. The mixture was then filtered, and the filtrate was extracted using 10% chloroform. After the evaporation of chloroform, tests were performed on the residue. Legal's test was conducted by adding sodium hydroxide and sodium nitroprusside to the extract. The appearance of a pink to blood-red coloration indicated the presence of cardiac glycosides.

#### Anthraquinone

Borntrager's test was carried out by adding 5 mL of chloroform to 0.5 g of the powdered sample. After shaking for five minutes, the mixture was filtered. An equal volume of 10% ammonia solution was added to the filtrate. The presence of free anthraquinones was confirmed by the appearance of a pink, red, or violet hue in the aqueous layer.

#### Alkaloids

The plant's alcoholic extract was vacuum-concentrated until completely dry. On a water bath, the dried extract was dissolved in 2N-hydrochloric acid, shaken, and filtered before being examined for alkaloids using various reagents. (1) In Wagner's test, 2 mL of the extract was treated with Wagner's reagent (a solution of iodine and potassium iodide). The formation of a reddish-brown precipitate indicated the presence of alkaloids. (2) In Dragendorff's test, a few drops of Dragendorff's reagent were added to the filtrate. A red precipitate formation confirmed the presence of alkaloids.

#### Steroids and terpenoids

A small amount of the extract was evaporated until completely dry. After dissolving the residue in two mL of chloroform, the filtrate was put through the following tests: (1) In the Salkowski reaction, concentrated sulfuric acid was carefully added down the side of the test tube containing 1 mL of the chloroform extract. A change in the yellow ring to a blood-red color indicated the presence of steroids and terpenes.

#### Coumarins

To detect coumarins, 1 g of the extract was placed in a test tube covered with filter paper moistened with diluted sodium hydroxide. The tube was heated in a water bath for a few minutes. Yellow fluorescence observed on the filter paper under UV light indicated the presence of coumarins.

#### Quinone

(1) NaOH test: Sodium hydroxide was added to the test substance. The appearance of a red or blue-green color indicated the presence of quinones.

#### Anthocyanin

2 mLof 2N HCl and ammonia were added to 2 mL of the aqueous extract. The formation of a pink-red color that changed to blue-violet indicated the presence of anthocyanins.

### Quantification of phenolic acid content

Using the Folin–Ciocalteau method, which was characterized by Chandra et al. (2014) and Mekky et al. (2024), the total phenolics in various parts of *S. marianum* were quantified. A 50 µL of the extract (1 mg/mL) or standard solution (0–50 μg/mL) was mixed with 0.2 mL of 0.5 M Folin–Ciocalteu reagent and 0.6 mL of distilled water, in triplicate. After 5 min of incubation, 1.0 mL of 8% (w/v) sodium carbonate solution was added. The mixture was then diluted to a final volume of 3.0 mL with distilled water and incubated in the dark for 30 min. Absorbance was measured at 760 nm using a UV–visible spectrophotometer. The total phenolic content in various parts of *S. marianum* was quantified based on a calibration curve generated using gallic acid as a standard (y = 0.003x + 0.0237, R^2^ = 0.9976). The results were reported as milligrams of gallic acid equivalents per gram of dry weight (mg GAE/g). Phenolic concentration was determined using the formula CV/m, where C represents the concentration in mg/mL, V is the volume of the extract in mL, and m denotes the sample mass in grams.

### Quantification of flavonoid content

Using a slight modification of the aluminium chloride calorimetric method first described by Aryal et al. (2019), we assessed the flavonoid content in various *S. marianum* parts. The amount of yellowish-orange coloration produced by the flavonoid-aluminium chloride complex reaction is a key variable in this process. A 500 μL of the extract (1 mg/mL) or quercetin standard solution (0–200 μg/mL) was mixed with 0.15 mL of 5% sodium nitrite (NaNO_2_) in triplicate. Then, 2 mL of distilled water was added, and the mixture was vortexed and left to stand for 5 min. Then, 0.15 mL of 10% aluminum chloride (AlCl_3_) was added, and the mixture was vortexed again and allowed to react for 6 min. Subsequently, 5 mL of distilled water and 2 mL of 4% sodium hydroxide (NaOH) were added. The final mixture was vortexed and incubated at 40 °C for 15 min. The absorbance was measured at 510 nm using a UV–visible spectrophotometer. The total flavonoid content was determined using the calibration curve of the quercetin standard (y = 0.0034x + 0.0135, R^2^ = 0.9971). Results were expressed as milligrams of quercetin equivalent per gram of dry weight (mg QE/g).

### Quantification of total tannin content

The total tannins were assessed using the Folin-Denis spectrophotometer technique by Makkar (2013) and Mahmoud et al. (2024). A 500 μL of each extract (1 mg/mL) was mixed with 100 mg of polyvinyl polypyrrolidone and diluted with 0.5 mL of DW. The tubes were incubated at 4 °C for 4 h, followed by centrifugation at 3000 rpm for 10 min at the same temperature. The supernatant contained the non-tannin phenolics. From this upper layer, 100 μL aliquots of the non-tannin phenolic fraction were taken in triplicate and mixed with 0.5 mL of 1 N Folin–Ciocalteu reagent. After 5 min of incubation, 2.5 mL of 5% sodium carbonate (Na_2_CO_3_) solution was added, including a blank sample. The mixture was vortexed and incubated in the dark for 40 min. Absorbance was measured at 725 nm using a UV–visible spectrophotometer. The tannin content was calculated using the standard calibration curve equation (y = 0.0086x + 0.0233, R^2^ = 0.9926). The results were expressed as milligrams of tannic acid equivalents per gram of dry sample (mg TAE/g), using the formula CV/m, where C is the concentration (mg/mL), V is the volume (mL), and m is the sample mass (g).

### Antimicrobial activity

Various pathogenic strains were tested to explore the antimicrobial potential of various parts of S. marianum extracts (stems, leaves, and flowers) that were extracted using different solvents. These included Gram-positive bacteria (*S aureus* ATCC 6538 and *B subtilis* ATCC 6633), Gram-negative bacteria (*P aeruginosa* ATCC 9027, *S typhimurium* ATCC 14028, and *E coli* ATCC 11229), and eukaryotic strains such as unicellular fungi (*C.albicans* ATCC 10231). To assess the effectiveness, the agar well diffusion method was used (Humphries et al. 2018). Every bacterium employed in this investigation was subcultured on Nutrient Agar (NA) and incubated at 37 °C for 48 h. The cultures were inoculated onto 15 cm diameter NA plates after being adjusted to the 0.5 McFarland turbidity standard. Each sample was separately diluted with DMSO to achieve the desired concentrations. A sterile cork borer made six wells on each agar plate, each measuring 0.6 mm in diameter. Each extract was then added to the wells in 100 µL.

Additionally, Ciprofloxacin was added in this experiment as a positive control. After an hour of refrigeration, for twenty-four hours, the plates were incubated at 35 ± 2 °C. Each well's surrounding clear zone's diameter was measured in millimetres. The experiment was performed in triplicate.

### Statistical analysis

The data was analyzed using an ANOVA, and Tukey's HSD was used to determine whether the mean difference between the treatments was significant at the *p* < 0.05 level. As in our earlier study, Minitab® version 18 (2017) was used for statistical analysis (Selim et al. 2024).

## Results

The present study aimed to investigate the phytochemical composition, nutritional value, and antimicrobial potential of different parts (stems, leaves, and flowers) of Silybum marianum L. using extracts obtained through various solvents. To achieve this, a series of experiments was conducted, starting with the determination of extractive yield and the visual characteristics (color) of the crude extracts. The assessment of proximate composition followed this to evaluate nutritional content, and preliminary phytochemical screening to identify the major secondary metabolites. Subsequently, quantitative analyses of total phenolics, flavonoids, and tannins were performed. Finally, the antimicrobial activities of the extracts were evaluated against a panel of Gram-positive and Gram-negative bacteria, as well as fungal strains. The detailed findings of these analyses are presented in the following subsections.

### Extractive yield and color of extracts

The color of the *S. marianum* extracts was obtained using petroleum ether, ethyl acetate, acetone, methanol, and water, which were used to differentiate the extracts from the various plant parts. The different parts displayed a range of extract colors, including green, dark green, brown-green, brown, and dark-brown, as shown in Table 1.

**Table 1:** Yield percentage and observed color of solvent extracts from stems, leaves, and flowers of *S. marianum*

Parts	Solvent used	Total hrs. of extraction	% Yield	Color of extract
	Petroleum ether	72 h	1.17 ± 0.15 ^i^	Dark green
	Ethyl acetate	72 h	1.64 ± 0.34 ^hi^	Green
Stems	Acetone	72 h	3.57 ± 0.21 ^fgh^	Brown
	Methanol	72 h	4.67 ± 0.38 ^efg^	Dark brown
	Water	72 h	6.33 ± 0.49 ^e^	Dark brown
	Petroleum ether	72 h	1.36 ± 0.13 ^i^	Dark green
	Ethyl acetate	72 h	2.59 ± 0.29 ^ghi^	Green
Leaves	Acetone	72 h	5.04 ± 0.26 ^ef^	Brown-green
	Methanol	72 h	5.43 ± 0.40 ^ef^	Brown
	Water	72 h	11.92 ± 1.42 ^d^	Dark brown
	Petroleum ether	72 h	3.90 ± 0.11 fg	Green
	Ethyl acetate	72 h	13.60 ± 1.01 cd	Green
Flowers	Acetone	72 h	15.30 ± 0.43 ^c^	Brown
	Methanol	72 h	20.07 ± 0.52 ^b^	Brown
	Water	72 h	24.83 ± 1.84 ^a^	Dark brown
Statics	S.S	2281.35		
	M.S	30.614		
	Df	8		
	*F*	58.76		
	*P*-value	0.001		

Data in the columns are presented as mean ± standard deviation. Values with different superscript letters indicate statistically significant differences based on Tukey's HSD test (*p* > 0.05). Mean square (M.S.), the sum of squares (S.S.), F-value (f), and degrees of freedom (Df) are also reported. Superscript letters (a, b, c, d, e, f, g, h, i) denote statistically significant differences between groups in pairwise comparisons

The yield obtained from the extraction of different parts of *S. marianum* using various solvents was calculated and presented in Table 1. The results showed that the flowers of *S. marianum* yielded the highest extract, followed by the leaves and stems, respectively. Among the solvents used, water extracts produced the highest percentage of yield, followed by methanol, acetone, ethyl acetate, and petroleum ether.

### Proximate composition

The proximate composition of *S. marianum* stems, leaves, and flowers is presented in Table 2. The leaves' moisture content was the highest (11.53%) and lowest (7.67%) in the flowers. The stems, leaves, and flowers that are produced from them should have outstanding shelf stability, a firm consistency, and a long shelf life due to their comparatively low moisture content. The flowers exhibit a higher lipid content (5.17%), followed by leaves (2.67%), and the weakest in stems (1.67%). It is worth mentioning that the stems gave the highest significant increase in ash content (28.67%) compared to leaves and flowers (16.50% and 7.27%), respectively.

**Table 2:** Proximate composition (moisture, ash, protein, lipid, nutritive value, carbohydrates) of dried stems, leaves, and flowers of *S. marianum*

Components	Stems	Leaves	Flowers	*P* value
Moisture (%)	10.13 ± 0.81 ^a^	11.53 ± 0.42 ^a^	7.67 ± 1.11 ^b^	0.003
Lipid (%)	1.67 ± 0.29 ^b^	2.67 ± 0.28 ^b^	5.17 ± 1.26 ^a^	0.004
Ash (%)	28.67 ± 2.08 ^a^	16.50 ± 2.60 ^b^	7.27 ± 1.57 ^c^	0.001
Protein (%)	6.95 ± 0.55 ^b^	9.15 ± 0.59 ^a^	10.03 ± 0.36 ^a^	0.001
Carbohydrate (%)	52.58 ± 7.08 ^b^	60.75 ± 2.41 ^ab^	69.86 ± 5.68 ^a^	0.022
Nutritive value (Kcal /100 g)	253.09 ± 24.96 ^b^	301.17 ± 5.51^b^	366.07 ± 31.38 ^a^	0.003

The results are mean ± SD, (n = 3). A significant difference (*P* < 0.05) is indicated by different letters on the same row. If pairwise comparisons are statistically different, they are marked by the superscripts a, b, and c

The crude protein level was significantly (*p* < 0.05) higher in the flowers (10.03%) compared to the leaves and stems, which had levels of 9.15% and 6.95%, respectively.

Carbohydrates are compounds produced during photosynthesis. Plants serve two primary functions: first, they provide building blocks for structural components like cellulose, which is crucial for cell wall formation; second, they act as energy sources to support plant growth. Various parts of the plant under study showed a significant amount of carbohydrates. Total carbohydrates reached their maximum value in flowers (69.86%) and a lower percentage in stems (52.58%), which agrees with protein and lipid content. The nutritive value was determined by multiplying the protein, fat, and carbohydrate values by 4.00, 9.00, and 4.00, respectively, and then summing the results. The nutritional value was significantly (*p* < 0.05) increased in flowers (366.07 kcal/ 100g) compared to leaves and stems (301.17 kcal/ 100g and 253.09 kcal/ 100g), respectively.

### Preliminary phytochemical screening

Phytochemicals play a crucial role in treating various diseases and continue to be used in traditional and modern medicinal practices. The qualitative chemical tests provide multiple insights into the types of phytochemical components found in the crude medication. Qualitative analysis of the phytochemicals revealed the presence of various phytoconstituents in the different dried parts of *S. marianum.*

Table 1S (supplementary) presents an overview of the findings from the qualitative phytochemical screening process conducted in different parts to identify the secondary metabolites of *S. marianum.* Alkaloids, flavonoids, tannins, glycosides, steroids, quinones, phenols, anthraquinones, cardiac glycosides, and terpenoids were found by phytochemical analysis of *S. marianum* in different parts. At the same time, saponins and anthocyanins were completely absent in all parts. On the other hand, coumarins are present in leaves and flowers and are completely lacking in stems (Fouda et al. 2022).

### Total phenolic acid content

**Fig. 2:**
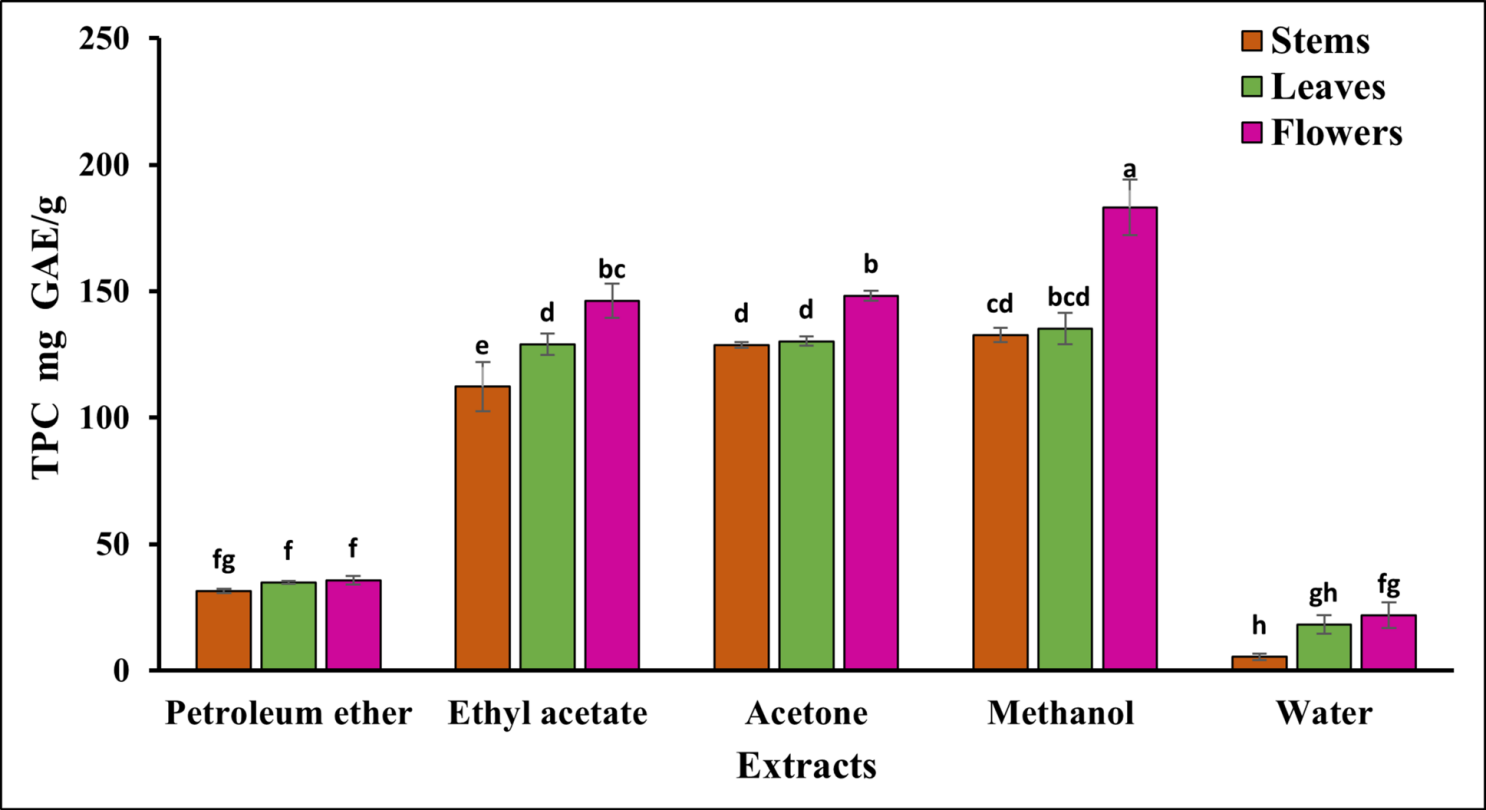
Total phenolic content. The values are mean ± SD from three replicates. Superscript letters (a, b, c, d, e, f, g, h) indicate statistically significant differences between groups in pairwise comparisons

The results showed that the total phenolic acid content varied across different plant parts (flowers > leaves > stems) and between extracts (methanol > acetone > ethyl acetate > petroleum ether > water). The solvent's polarity, which has been demonstrated to be essential in improving the solubility of phenolics, affected the total phenolic content of the various extracts (Fig. 2). Table 2S (supplementary) revealed that the methanol extract from the flowers had the highest phenol content (183.12 ± 11.02 mg GAE/g). In Comparison, the water extract from the stems contained the lowest amount (5.45 ± 1.32 mg GAE/g).

### Total flavonoid content

**Fig. 3:**
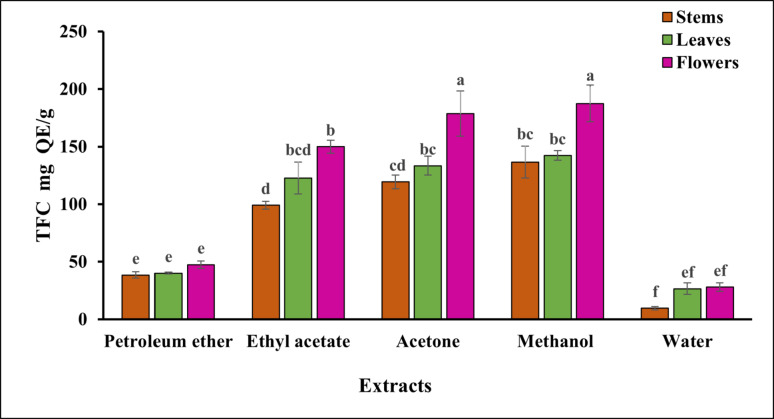
The total flavonoid content of each extract was measured in various parts of *S. marianum* using different solvent fractions in standard equivalents. The values represent the mean ± standard deviation of three replicates. Superscripts a, b, c, d, e, and f indicate pairwise comparisons and denote statistical differences

Quercetin equivalents were used to express the flavonoid content, which was calculated using a standard quercetin. The total quantity of flavonoids was highest in the methanolic extract (Fig. 3), with the flowers showing the highest levels of flavonoids compared to the leaves and stems. Table 2S (supplementary) revealed that the methanol fraction from the flowers had a significantly higher flavonoid content (187.43 ± 15.91 mg QE/g) compared to the other solvent fractions (*P* < 0.05). In contrast, the water extracted from the stems had the lowest value, 9.60 ± 1.5 mg QE/g. No significant difference was observed between the methanol and acetone extracts of the flowers, and between the stems, leaves, and flowers of *S. marianum* in the petroleum ether extract (*P* > 0.05).

### Total tannin content

**Fig. 4:**
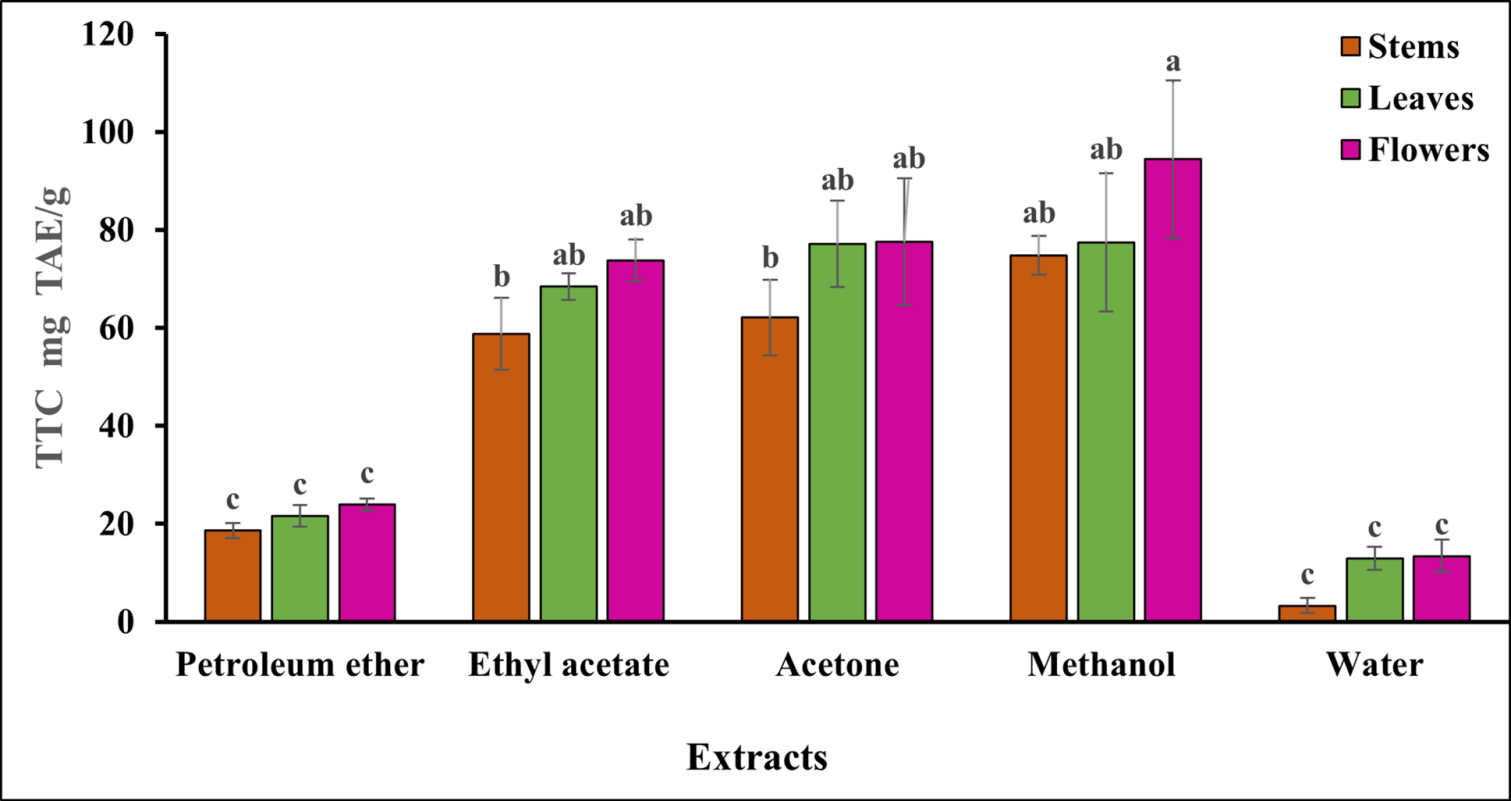
Total tannin content, expressed as mg TAE (tannic acid equivalents) per g of each extract, was measured in various parts of *S. marianum* using different solvent fractions in standard equivalents. The values represent the mean ± standard deviation of three replicates. Superscripts a, b, and c indicate pairwise comparisons and denote statistical differences

The amount of tannic acid equivalent (mg TAE/g) per gram of extract was used to represent the total tannin content. The study results indicate that the methanol extract contains a higher percentage of total tannin (Fig. 4), with the flowers containing the highest levels of tannins compared to the leaves and stems. Table 2S (supplementary) also revealed that the methanol fraction from the flowers had a significantly higher tannin content (94.40 ± 16.04 mg TAE/g of extract) compared to the other solvent fractions (*P* < 0.05). In contrast, the water extract from the stems had the lowest value at 3.27 ± 1.53 mg TAE/g of extract. There was no noticeable difference between the acetone and ethyl acetate fractions in the leaves and flowers of *S. marianum* (*P* > 0.05), and no significant difference between the acetone and ethyl acetate fractions in stems (*P* > 0.05). Also, in the methanol fraction, there was no significant difference between leaves and stems (*P* > 0.05). On the other hand, in petroleum ether extract and water fractions, there was no significant difference between different parts of the plant under study (*P* > 0.05).

### Antimicrobial activity

The agar well diffusion method was used to analyze the performance of different parts of *S. marianum* extracts (stems, leaves, and flowers) that were extracted using various solvents in suppressing the development of numerous pathogenic microbes, such as Gram-positive bacteria (*S aureus* ATCC 6538 and *B. subtilis* ATCC 6633), Gram-negative bacteria (*P. aeruginosa* ATCC 9027, *S. typhimurium* ATCC 14028, and *E. coli* ATCC 11229), and eukaryotic strains such as unicellular fungi (*C. albicans* ATCC 10231).

**Fig. 5:**
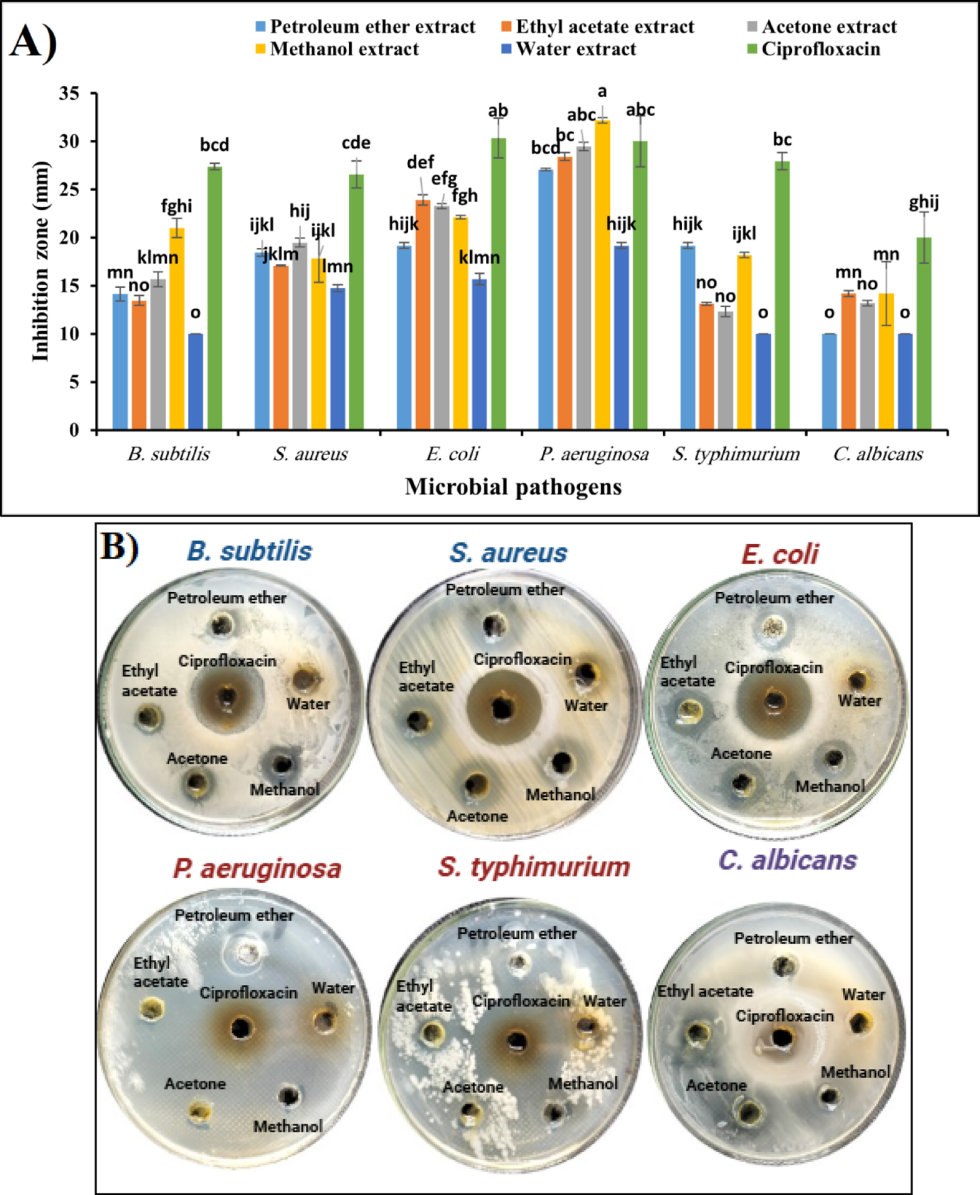
**A** Antimicrobial activity (inhibition zones, mm) of solvent extracts from *S. marianum* stems. Values are mean ± SD (n = 3); different superscripts indicate significant differences (*p* < *0.05*). **B** Representative agar well diffusion images showing the inhibitory effects of each extract compared to Ciprofloxacin

The data represented graphically in Fig. 5A and B summarize the antimicrobial activity of five different solvent extracts of *S. marianum* stems compared with Ciprofloxacin as a positive control.

The results shown in Fig. 5A and B demonstrated that the inhibition zones of petroleum ether extract of *S. marianum* stems were 14.13 ± 0.72, 18.43 ± 0.40, 19.16 ± 0.28, 27.06 ± 0.11, 19.16 ± 0.28, and 10 ± 0.0 mm for *B. subtilis, S aureus*, *E. coli, P. aeruginosa, S. typhimurium*, and *C. albicans,* respectively. On the other hand, the inhibition zones of ethyl acetate extract were 13 ± 0.51, 17.07 ± 0.05, 23.9 ± 0.55, 28.4 ± 0.4, 13.1 ± 0.1, and 14.17 ± 0.2 mm for *B. subtilis, S aureus*, *E coli, P aeruginosa, S. typhimurium*, and *C. albicans,* respectively. Moreover, the inhibition zones of acetone extract were 15.7 ± 0.8, 19.5 ± 0.5, 23.3 ± 0.3, 29.5 ± 0.5, 12.3 ± 0.5, and 13.2 ± 0.3 mm for *B. subtilis, S. aureus*, *E. coli, P. aeruginosa, S. typhimurium*, and *C. albicans,* respectively. Additionally, the effects of methanol extract were 21.0 ± 1, 17.83 ± 2.47, 22.1 ± 0.17, 32.16 ± 0.29, 18.16 ± 0.29, and 14.16 ± 3.33 mm for *B. subtilis, S. aureus*, *E. coli, P. aeruginosa, S. typhimurium*, and *C. albicans,* respectively. In contrast, the inhibition zones of water extract were 10 ± 0, 14.75 ± 0.35, 15.66 ± 0.57, 19.16 ± 0.28, 10 ± 0, and 10 ± 0 mm for *B. subtilis, S. aureus*, *E. coli, P. aeruginosa, S. typhimurium*, and *C. albicans,* respectively. Furthermore, the effects of Ciprofloxacin were 27.36 ± 0.32, 26.56 ± 1.40, 30.33 ± 2.08, 30 ± 2.64, 27.93 ± 0.90, and 20 ± 2.64 mm for *B. subtilis, S. aureus*, *E. coli, P. aeruginosa, S. typhimurium*, and *C. albicans,* respectively.

**Fig. 6:**
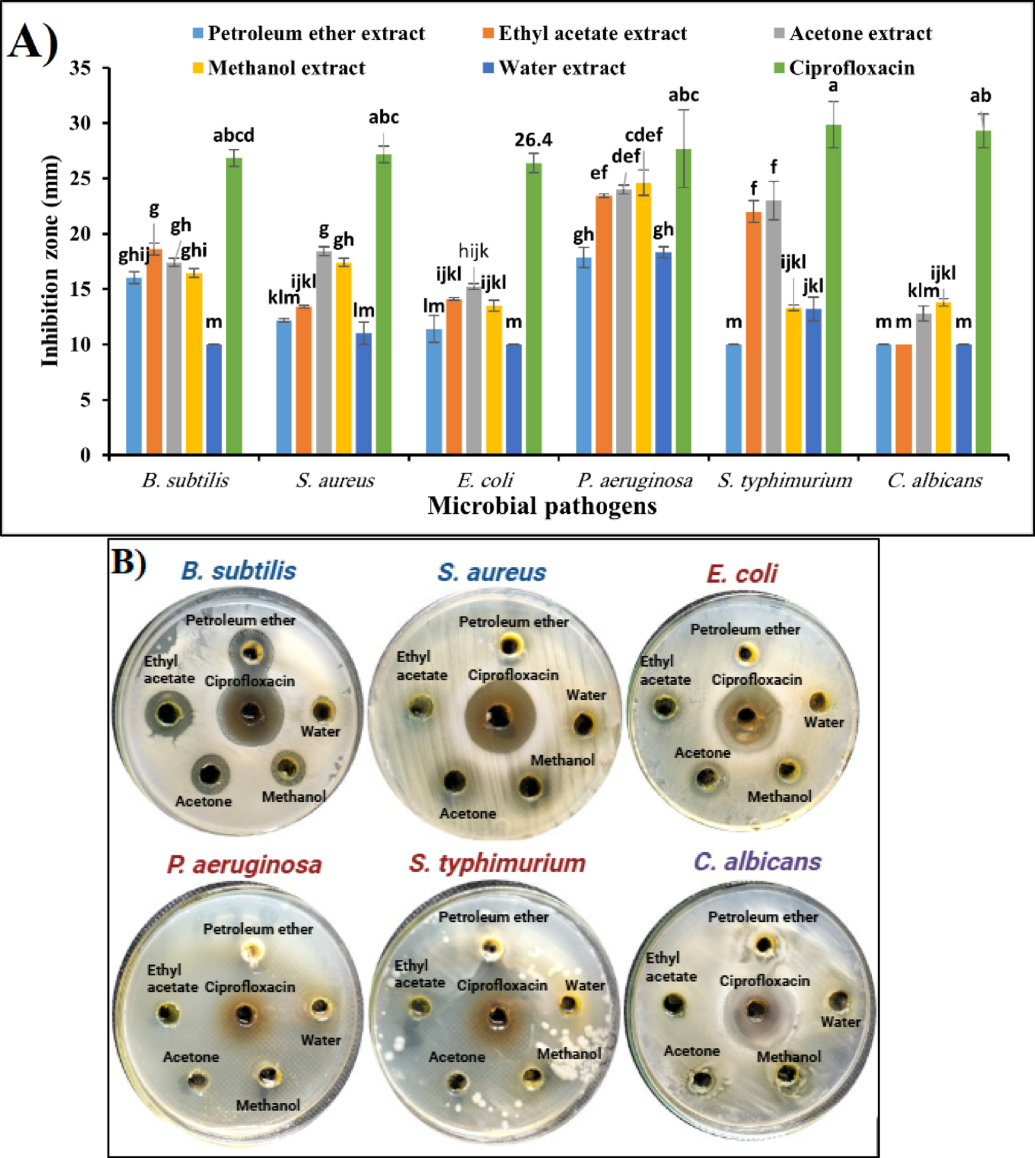
**A** Antimicrobial activity of solvent extracts from *S. marianum* leaves. Mean ± SD (n = 3); different superscripts denote significant differences (*p* < *0.05*). **B** Representative agar well diffusion images showing the inhibitory effects of each extract compared to Ciprofloxacin

On the other hand, the data represented graphically in Fig. 6A and B summarise the antimicrobial activity of five different solvent extracts of *S. marianum* leaves compared with Ciprofloxacin as a positive control. The results shown in Fig. 6A and B demonstrated that the inhibition zones of petroleum ether extract of *S. marianum* leaves were 16.03 ± 0.5, 12.17 ± 0.10, 11.4 ± 1.2, 17.87 ± 0.9, 10 ± 0.0, and 10 ± 0.0 mm for *B. subtilis, S. aureus*, *E. coli, P. aeruginosa, S. typhimurium*, and *C. albicans,* respectively. On the other hand, the inhibition zones of ethyl acetate extract were 18.6 ± 0.51, 13.4 ± 0.1, 14.1 ± 0.1, 23.4 ± 0.1, 22 ± 1, and 10 ± 0 mm for *B. subtilis, S. aureus*, *E. coli, P. aeruginosa, S. typhimurium*, and *C. albicans,* respectively. Moreover, the inhibition zones of acetone extract were 17.4 ± 0.3, 18.4 ± 0.4, 15.2 ± 0.2, 24 ± 0.4, 23 ± 1.7, and 12.8 ± 0.6 mm for *B. subtilis, S. aureus*, *E. coli, P. aeruginosa, S. typhimurium*, and *C. albicans,* respectively. Additionally, the effects of methanol extract were 16.4 ± 0.4, 17.4 ± 0.3, 13.5 ± 0.5, 24.6 ± 1.15, 13.3 ± 0.20, and 13.8 ± 0.3 mm for *B. subtilis, S. aureus*, *E. coli, P. aeruginosa, S. typhimurium*, and *C. albicans,* respectively. At the same time, the inhibition zones of water extract were 10 ± 0, 11 ± 1, 10 ± 0, 18.3 ± 0.5, 13.2 ± 1.05, and 10 ± 0 mm for *B. subtilis, S. aureus*, *E. coli, P. aeruginosa, S. typhimurium*, and *C. albicans,* respectively. Furthermore, the effects of Ciprofloxacin were 26.8 ± 0.76, 27.1 ± 0.76, 26.4 ± 0.87, 27.66 ± 3.5, 29.86 ± 2.1, and 29.3 ± 1.53 mm for *B. subtilis, S. aureus*, *E. coli, P. aeruginosa, S. typhimurium*, and *C. albicans,* respectively.

**Fig. 7:**
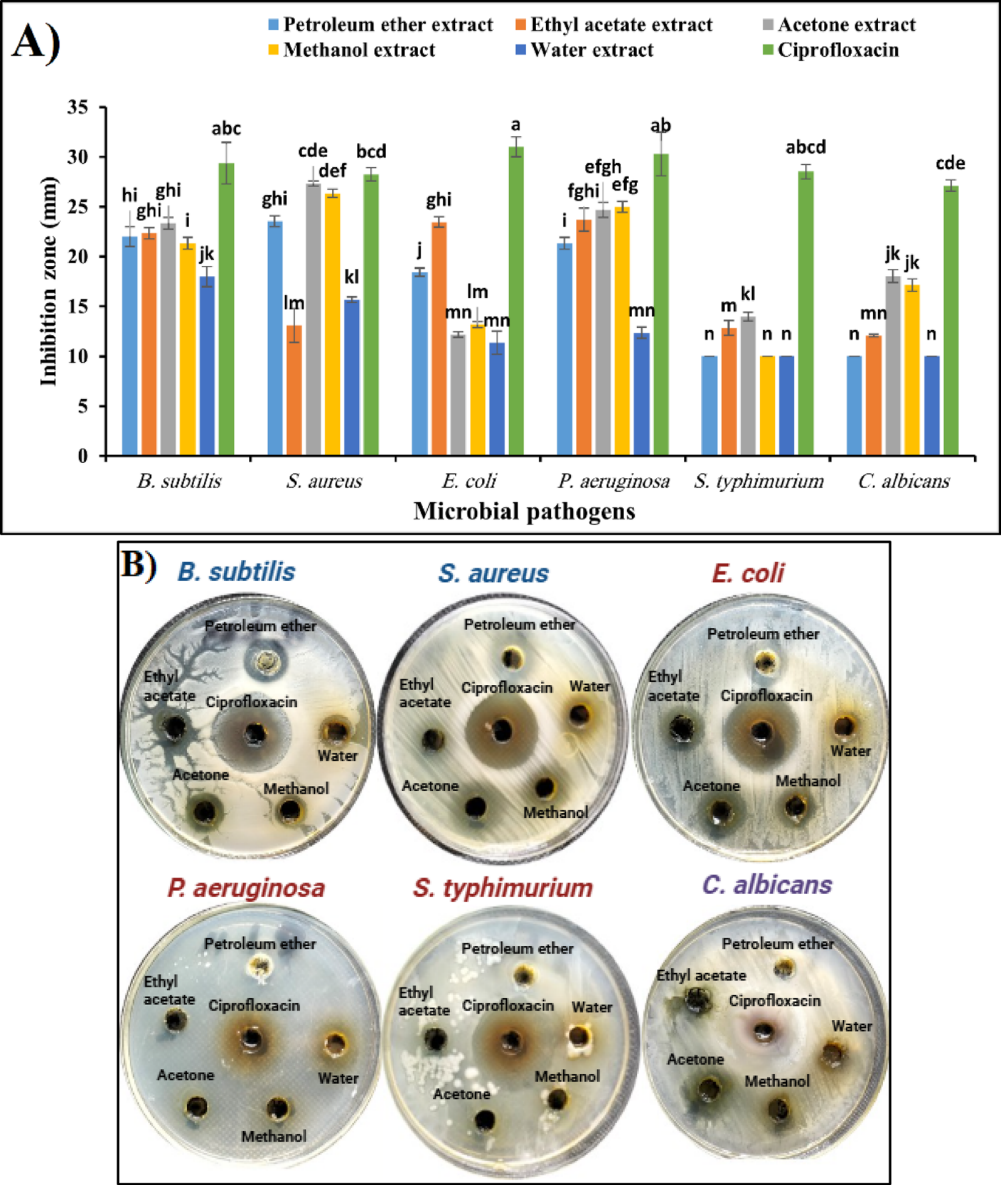
**A** Antimicrobial activity of solvent extracts from *S. marianum* flowers. Mean ± SD (n = 3); different superscripts indicate significant differences (*p* < *0.05*). **B** Representative agar well diffusion images showing the inhibitory effects of each extract compared to Ciprofloxacin

Furthermore, the data represented graphically in Fig. 7A and B summarise the antimicrobial activity of five different solvent extracts of *S. marianum* flowers compared with Ciprofloxacin as a positive control.

The results shown in Fig. 7A and B demonstrated that the inhibition zones of petroleum ether extract of *S. marianum* flowers were 22 ± 1, 23.53 ± 0.55, 18.43 ± 0.4, 21.33 ± 0.57, 10 ± 0.0, and 10 ± 0.0 mm for *B. subtilis, S. aureus*, *E. coli, P. aeruginosa, S. typhimurium*, and *C. albicans,* respectively. On the other hand, the inhibition zones of ethyl acetate extract were 22.3 ± 0.57, 13.1 ± 1.6, 23.4 ± 0.5, 23.7 ± 1.15, 12.8 ± 0.7, and 12.1 ± 0.11 mm for *B. subtilis, S. aureus*, *E. coli, P. aeruginosa, S. typhimurium*, and *C. albicans,* respectively. Moreover, the inhibition zones of acetone extract were 23.3 ± 0.6, 27.3 ± 0.3, 12.2 ± 0.3, 24.7 ± 0.8, 14 ± 0.5, and 18 ± 0.7 mm for *B. subtilis, S. aureus*, *E. coli, P. aeruginosa, S. typhimurium*, and *C. albicans,* respectively. Additionally, the effects of methanol extract were 21.33 ± 0.58, 26.33 ± 0.42, 13.1 ± 0.29, 24.96 ± 0.55, 10 ± 0.0, and 17.13 ± 0.61 mm for *B. subtilis, S. aureus*, *E. coli, P. aeruginosa, S. typhimurium*, and *C. albicans,* respectively. At the same time, the inhibition zones of water extract were 18 ± 1, 15.66 ± 0.2, 11.33 ± 1.1, 12.33 ± 0.57, 10 ± 0, and 10 ± 0 mm for *B. subtilis, S. aureus*, *E. coli, P. aeruginosa, S. typhimurium*, and *C. albicans,* respectively. Furthermore, the effects of Ciprofloxacin were 29.33 ± 0.2, 28.2 ± 0. 6, 31 ± 1, 30.2 ± 2.1, 28.5 ± 0.7, and 27.1 ± 0.55 mm for *B. subtilis, S. aureus*, *E. coli, P. aeruginosa, S. typhimurium*, and *C. albicans,* respectively.

## Discussion

Different parts of *S. marianum* extracts showed various extract colors (Table 1). The results were consistent with those previously reported for *Centaurea calcitrapa* L. (Asteraceae family), where the aerial flowering parts were dissolved in methanol, ethyl acetate, chloroform, and aqueous solutions. They showed different extract colors, such as yellow–brown, green, dark green, and dark brown (Mekky et al. 2024).

The extractive yield was directly influenced by the type of solvent used and its extraction efficiency. Factors like solvent volume, energy usage, extraction duration, and their effects on the environment and human health are all related to extraction efficiency (Azmir et al. 2013). Therefore, choosing an economical and environmentally friendly solvent is critical for separating bioactive phytochemicals from plant materials. This study used three different parts of the S. marianum stems, leaves, and flowers to extract bioactive compounds from dried plant materials, along with solvents with different polarities: petroleum ether, ethyl acetate, acetone, methanol, and water.

The water and methanol have higher dielectric constants than acetone, ethyl acetate, and petroleum ether due to their polar protic nature. These properties facilitate the solubilization of polar and some non-polar secondary metabolites to a greater extent in the former solvents than in the latter (Kalia et al. 2008). The polarity of specific compounds found in plant parts is thought to be responsible for the variance in extractable matter yield in different solvents. This suggests that both internal and external influences may impact the origin of the bioactive principle of medicinal plants (Hayouni et al. 2007). High extractive values that are soluble in water and alcohol disclose the existence of polar substances such as glycosides, tannins, and phenols, as documented in the literature on secondary metabolites (Jones and Kinghorn 2012; Nandhakumar and Indumathi 2013). Similar patterns can be seen in the percentage extractive yield results of solvent extraction of *Centauraea calcitrapa* L. aerial flowering parts (methanol-11.24% > water-9.09% > ethyl acetate-3.02% > chloroform-2.96%) were reported by Mekky et al., (2024).

The enhanced antimicrobial efficacy observed in methanolic and aqueous extracts can be attributed to their superior ability to extract polar phytochemicals, particularly phenolic acids and flavonoids, which are known for their broad-spectrum antimicrobial activities. Phenolics contain multiple hydroxyl groups, making them highly soluble in polar solvents due to their ability to form hydrogen bonds. This polarity-based selectivity explains polar extracts' elevated total phenolic and flavonoid content, as confirmed in our phytochemical analysis. These bioactives exert antimicrobial effects through membrane disruption, metal ion chelation, enzyme inhibition, and oxidative stress induction within microbial cells. Thus, the observed trend supports the hypothesis that solvent polarity directly influences the yield of antimicrobial phytochemicals, and consequently, the antimicrobial potential of plant extracts (Aldayel 2023; El-Sapagh et al. 2023; Chikhoune et al. 2024).

An effective extraction technique should yield high amounts of extract while minimizing changes to the functional properties of the desired compounds (Quispe-Condori et al. 2008). The biological activities of extracts made with various extraction methods have been found to differ in several studies. As a result, it is crucial to choose the right extraction technique and solvent depending on the sample matrix's properties, the analytes' chemical makeup, the interactions between the matrix and analytes, extraction efficiency, and the desired extract properties (Hayouni et al. 2007; Ishida et al. 2001).

Low moisture content reduces the likelihood of microbial growth, undesirable fermentation, early seed germination, and other undesirable biochemical changes frequently associated with these processes (Kaleta and Górnicki 2013). The lipid content of *S. marianum* was higher compared to the leaves of *Fagonia cretica*, *Pisum sativum*, and *Brassica oleracea* (Imran et al. 2007), and like that of wheat (2.83 g 100g^−1^ DW). The lipid content in the family Asteraceae is generally less than 4% (Achika et al. 2014). The differences are likely related to factors such as plant growth stages, climate, and geography.

Ash content reflects the amount of mineral elements present in the plant(Biel et al. 2020). Ash value is one of the common characteristics among the numerous physicochemical characteristics analyzed in this study. It is used to identify and determine the purity of the plant material, especially when it is in powder form for future research or application. The ash values reveal the presence of various impurities, including silicate, oxalate, phosphate, and carbonate. These impurities might originate from plants (natural or physiological ash) or external materials, including sand and soil that sticks to plant surfaces (non-physiological ash) (Kadam et al. 2012). This may be related to increased total ion accumulation due to increased soil moisture stress and soil salinity, which corresponded with the results reported by Larcher (1996). A high ash content is useful for assessing the quality and grading of the plant, indicating the mineral content in the sample (Smith 2009).

Sufficient protein concentration in plants can facilitate the synthesis of hormones that regulate several physiological processes, including growth, tissue repair, and maintenance of body protein (Charalambous 1993). It also indicates the potential benefit of *S. marianum* because proteins are essential for synthesizing body tissues and regulated substances such as hormones and enzymes (Vaughan and Judd 2003). The increase in carbohydrates is due to the rise in soil moisture in the studied habitat, which increases the accumulation of carbohydrates remarkably. These results are consistent with those reported by Escudero et al. (2003).

The assay for phytochemical screening is a rapid and low-cost method for identifying bioactive ingredients. *S. marianum* contains bioactive chemicals that are multi-constituent combinations with distinct solvents; nonetheless, identifying and separating them can still be challenging. Virtually, most of them must be purified by a combination of various purification and chromatographic techniques to isolate the bioactive compounds (Hamburger and Hostettmann 1991; Hostettmann et al. 1997). Plants with various bioactive components imported for medical purposes may be used to treat serious pathogenic diseases in humans. Phytochemicals can be divided into two categories: primary and secondary substances. Amino acids, proteins, sugar, and chlorophyll are the primary constituents. Terpenoids and alkaloids are examples of secondary compounds (Wadood 2013). These substances act as a defence for plants against insects, herbivores, and microbes. For instance, the most basic phytochemicals that are helpful against viruses are called phenolics (Wild 1994) and fungi (Duke 2002).

Because phenols contain hydroxyl groups that function as hydrogen donors, they are known to have potent antioxidant properties. Due to their redox properties, they function as reducing agents and scavengers of free radicals (Afolayan and Wintola 2011). The amount of GAE per g of the sample was used in this investigation to determine the phenolic content. The results confirmed that the solvents varied in their ability to extract phenols from the sample. Notably, phenolic compounds are often linked to various biomolecules (such as proteins, polysaccharides, chlorophyll, terpenes, and inorganic compounds). Therefore, the choice of solvent for extracting a particular class or group of compounds should be based on the structural characteristics and solubility of the target molecule (Ghasemzadeh et al. 2011).

The current study's findings are consistent with those of multiple researchers who have proposed a connection between antioxidant activity and phenolic compounds (Cai et al. 2004; Zheng and Wang 2001). Likewise, Tawaha et al. (2007) revealed a relationship between *S. marianum* phenolic content and antioxidant activity. The effectiveness of free radical scavenging varies among plant species, according to numerous studies on medicinally significant plants. Hădărugă and Hădărugă, (2009) also noted comparable antioxidant activity for *S. marianum* essential oil constituents. Additionally, the proximate analysis showed high fat, carbohydrate, protein, mineral, and ash content.

Flavonoids, secondary metabolites produced by long-term natural selection, are found in high concentrations in the roots, stems, leaves, flowers, and fruits of higher plants, including those in the *Rutaceae, Lamiaceae, Fabaceae, Apiaceae, Ginkgoaceae*, and *Asteraceae* families (Roy et al. 2022). As oxidizing and microbiological agents, these substances give leaves, flowers, and fruits their color and are essential to plants' defence (Shen et al. 2022). Both natural and synthetic flavonoids have been shown in numerous studies to have both individual and combined antimicrobial activity against drug-resistant fungi (Jin 2019), bacteria (Xie et al. 2015), and viruses (Badshah et al. 2021). The present study observed that the effect of different solvents on flavonoid content mirrors their effect on phenolic content. Several studies have reported varying levels of flavonoid content in *S. marianum* (Al-Obaidi et al. 2017; Ghafor et al. 2014; Kachel et al. 2023; Shah et al. 2011).

Secondary compounds with a wide range of chemical structures, and tannins are abundant in plants and can be categorised into two main types: hydrolyzable tannins and condensed tannins. Due to their capacity for neutralizing free radicals, hydrolyzable and condensed tannins have great promise as biological antioxidants. Tannic acid's antioxidant action is due to its polyphenolic nature. Tannin is useful as an antidote for heavy metal and alkaloid poisoning, in addition to its antiviral and antibacterial properties. Thanks to its powerful antioxidant properties, it can neutralize the body's superoxide free radicals and stop aging(Shen et al. 2022). Again, the methanol extract contains a higher percentage of total tannin (Fig. 4), with the flowers containing the highest levels of tannins compared to the leaves and stems. Therefore, the methanol extracts were the solvent of choice for tannin extraction because they showed a significantly greater difference in amount than the other solvent extracts (*P* < 0.05).

The comparative analysis of different plant parts, stems, leaves, and flowers provides valuable insights for several reasons. Firstly, the concentration and diversity of secondary metabolites (e.g., polyphenols, flavonoids, tannins, alkaloids) vary significantly among different parts of the *S. marianum* plant due to their distinct physiological roles and metabolic profiles. For example, flowers often accumulate higher levels of flavonoids and phenolic compounds involved in pigmentation and protection against oxidative stress, leaves may contain higher photosynthesis-related metabolites, and stems may have structural or defence-related compounds (Javeed et al. 2022).

The second reason is that different solvents have varying boiling points and polarities, which affect their ability to extract classes of phytochemicals from each plant part. For instance, Aqueous ethanol (a polar solvent with a moderate boiling point) was more effective at extracting polyphenols and flavonoids, particularly from leaves and flowers, where these compounds are more abundant and soluble. In contrast, non-polar solvents (e.g., hexane) showed limited efficiency, mainly extracting lipophilic compounds from stems, with lower antimicrobial activity. This highlights the importance of matching solvent polarity with metabolite polarity and recognising that different plant tissues yield optimal results with varying solvent systems (Abubakar and Haque 2020).

The third reason is that our findings revealed clear differences in extraction yields and compound concentration across plant parts. For instance, leaf extracts showed higher total phenolic and flavonoid content than stems, which had lower concentrations and lower antimicrobial inhibition zones. These differences help in targeted selection of plant parts for specific therapeutic or nutraceutical applications (Iraqi et al. 2025).

The fourth reason, as reported in our study, is that there are also notable differences in nutritional composition among plant parts. Leaves and flowers had higher content of bioactive compounds with nutritional and health-promoting potential, which enhances their value in functional food or dietary supplement development.

The rising prevalence of infections brought on by pathogens, including *P. aeruginosa, S. aureus, E. coli,* and *C. albicans*, causes the rise in infection morbidity rates globally (Rakelly de Oliveira et al. 2015). Antibiotic resistance and a lack of antimicrobial supplies, particularly in developing nations, are some of the factors contributing to this rise. Therefore, using plant extracts to treat microbial illnesses has grown in popularity over the past few decades (Bibi et al. 2011). The antimicrobial efficacy of milk thistle extracts against various microorganisms highlights its potential as a natural antimicrobial agent. The presence of bioactive compounds like silymarin, flavonoids, and phenolic acids in milk thistle is likely responsible for its antimicrobial properties (Rawash et al. 2024).

The antimicrobial activity results of five different solvent extracts of *S. marianum* stems were compared with Ciprofloxacin as a positive control (Fig. 5A and B). The inhibition zones for each extract varied depending on the bacterial and unicellular fungi strains tested.

In Comparison of solvent extracts, petroleum ether extract showed moderate activity against *B. subtilis* (14.13 mm), *S. aureus* (18.43 mm), *E. coli* (19.16 mm), *S. typhimurium* (19.16 mm), and *P. aeruginosa* (27.06 mm). In contrast, Lower inhibition zones against *C. albicans* (10 mm). Also, it demonstrated high efficacy against P. aeruginosa but was weak against *C. albicans*. On the other hand, ethyl acetate extract is generally better than ethyl acetate extract, with high efficacy against *E. coli* (23.9 mm), *P. aeruginosa* (28.4 mm), and *C. albicans* (14.17 mm). Additionally, moderate activity against *B. subtilis* (13 mm), *S. aureus* (17.07 mm), and *S. typhimurium* (13.1 mm). Furthermore, the acetone extract showed high activity, particularly against *P. aeruginosa* (29.5 mm) and *E. coli* (23.3 mm). Also effective against *B. subtilis* (15.7 mm) and *S. aureus* (19.5 mm). While moderate inhibition of *S. typhimurium* (12.3 mm) and *C. albicans* (13.2 mm). This extract is one of the most effective, especially against *P. aeruginosa*. Additionally, methanol extract exhibited the highest activity among all extracts, with inhibition zones of *B. subtilis* (21 mm), *S. aureus* (17.83 mm), *E. coli* (22.1 mm), *P. aeruginosa* (32.16 mm), *S. typhimurium* (18.16 mm), and *C. albicans* (14.16 mm). While the highest inhibition against *P. aeruginosa* (32.16 mm) is comparable to Ciprofloxacin (30 mm). Also, the strongest extract indicates that methanol is the most effective solvent for extracting antimicrobial compounds from *S. marianum* stems. In contrast, the water extract displayed the weakest activity, with inhibition zones ranging from 10 mm to 19.16 mm. Lowest efficacy against *B. subtilis* (10 mm), *S. typhimurium* (10 mm), and *C. albicans* (10 mm).

In Comparison with Ciprofloxacin (Positive Control), Ciprofloxacin exhibited the highest inhibition zones across all strains, ranging from 20 mm (*C. albicans*) to 30.33 mm (*E. coli*). None of the extracts outperformed Ciprofloxacin, but the methanol extract came close, especially against *P. aeruginosa* (32.16 mm vs. 30 mm for Ciprofloxacin). The acetone and ethyl acetate extracts also showed notable antimicrobial activity, but remained lower than Ciprofloxacin.

In conclusion, methanol is the most effective solvent for extracting antimicrobial compounds from *S. marianum* stems. Although the acetone and ethyl acetate extracts were less effective than Ciprofloxacin, they also demonstrated significant antibacterial activity. Water extract had the weakest antimicrobial effect, suggesting that water is not a suitable solvent for extracting bioactive compounds. While Ciprofloxacin remains the most effective antimicrobial agent, the results indicate that *S. marianum* stem extracts, especially methanol and acetone extracts, have promising antibacterial potential.

On the other hand, the antimicrobial activity of five different solvent extracts of *S. marianum* leaves was evaluated against six microbial strains, using Ciprofloxacin as a positive control (Fig. 6A and B). In Comparison of solvent extracts, petroleum ether extract showed moderate activity against *B. subtilis* (16.03 mm) and *P. aeruginosa* (17.87 mm). Also, weak activity against *E. coli* (11.4 mm) and *S. aureus* (12.17 mm), while no significant inhibition against *S. typhimurium* and *C. albicans* (both 10 mm). Additionally, ethyl acetate extract exhibited improved activity against *B. subtilis* (18.6 mm) and *P. aeruginosa* (23.4 mm). It has moderate effectiveness against *S. aureus* (13.4 mm) and *E. coli* (14.1 mm), while showing strong inhibition against *S. typhimurium* (22 mm), but was ineffective against *C. albicans* (10 mm). Furthermore, the Acetone Extract showed broad-spectrum activity with higher inhibition zones, particularly against *P. aeruginosa* (24 mm) and *S. typhimurium* (23 mm). Also, moderate activity against *B. subtilis* (17.4 mm) and *S. aureus* (18.4 mm), while slightly lower activity against *E. coli* (15.2 mm) and *C. albicans* (12.8 mm). On the other hand, methanol extracts demonstrated significant inhibition against *P. aeruginosa* (24.6 mm) with moderate inhibition for *B. subtilis* (16.4 mm), *S. aureus* (17.4 mm), and *E. coli* (13.5 mm), while showing lower effectiveness against *S. typhimurium* (13.3 mm) and *C. albicans* (13.8 mm). In contrast, water extract exhibited the weakest antimicrobial activity across all tested strains, while moderate activity was only against *P. aeruginosa* (18.3 mm) and *S. typhimurium* (13.2 mm). Also, there is limited or no activity against the remaining microorganisms.

In Comparison with Ciprofloxacin (Positive Control), Ciprofloxacin exhibited significantly higher inhibition zones across all microbial strains (ranging from 26.4 mm to 29.86 mm), confirming its superior antimicrobial activity compared to all *S. marianum* leaf extracts.

In conclusion, acetone and ethyl acetate extracts were the most effective, especially against *P. aeruginosa* and *S. typhimurium*. Methanol extract also showed good antimicrobial properties but was slightly less effective than acetone and ethyl acetate extracts. Water extract had the lowest activity, suggesting that active antimicrobial compounds in *S. marianum* leaves may be more soluble in organic solvents. Ciprofloxacin remains the most potent antimicrobial agent in Comparison, highlighting the limitations of natural extracts for clinical applications. These findings suggest that organic solvent extracts, particularly acetone and ethyl acetate, could be promising candidates for further antimicrobial research and potential pharmaceutical applications.

The antimicrobial activity of five different solvent extracts from *S. marianum* flowers (Fig. 7A and B) was assessed against six microbial strains (*B. subtilis, S. aureus, E. coli, P. aeruginosa, S. typhimurium*, and *C. albicans*), with Ciprofloxacin serving as a positive control. The inhibition zones measured in millimetres reflect the relative effectiveness of each extract in inhibiting microbial growth.

In Comparison of solvent extracts, petroleum ether extract exhibited strong activity against *B. subtilis* (22 mm), *S. aureus* (23.53 mm), and *P. aeruginosa* (21.33 mm), with moderate inhibition of *E. coli* (18.43 mm), and was Ineffective against *S. typhimurium* and *C. albicans* (both 10 mm). On the other hand, ethyl acetate showed broad-spectrum activity, with notable inhibition against *E. coli* (23.4 mm) and *P. aeruginosa* (23.7 mm). Also, high activity against *B. subtilis* (22.3 mm), while lower inhibition against *S. aureus* (13.1 mm), *S. typhimurium* (12.8 mm), and *C. albicans* (12.1 mm). Furthermore, acetone extract demonstrated the highest inhibition against *S. aureus* (27.3 mm) and *P. aeruginosa* (24.7 mm), strong inhibition against *B. subtilis* (23.3 mm), and Weak inhibition of *E. coli* (12.2 mm) but better activity against *S. typhimurium* (14 mm) and *C. albicans* (18 mm). Additionally, Methanol Extract showed good activity against *S. aureus* (26.33 mm) and *P. aeruginosa* (24.96 mm) with moderate inhibition of *B. subtilis* (21.33 mm) and *E. coli* (13.1 mm), and limited activity against *S. typhimurium* (10 mm) but better inhibition of *C. albicans* (17.13 mm). In contrast, Water Extract had the lowest overall activity with Moderate inhibition of *B. subtilis* (18 mm) and *S. aureus* (15.66 mm) and Weak or negligible effects against *E. coli, P. aeruginosa, S. typhimurium,* and *C. albicans* (≤ 12.33 mm).

In Comparison with Ciprofloxacin (Positive Control), Ciprofloxacin exhibited the highest antimicrobial activity, with inhibition zones ranging from 27.1 mm to 31 mm across all tested strains. This confirms its superior efficacy compared to all *S. marianum* flower extracts.

In conclusion, Acetone extract was the most effective against *S. aureus, P. aeruginosa,* and *C. albicans*. Petroleum ether extract and ethyl acetate extracts also exhibited good antimicrobial properties, especially against *B. subtilis, S. aureus,* and *E. coli*. Methanol extract had moderate activity but was particularly effective against *S. aureus* and *C. albicans*. Water extract was the least effective, highlighting the poor solubility of active antimicrobial compounds in water. The results suggest that *S. marianum* flower extracts, particularly those from acetone, petroleum ether extract, and ethyl acetate solvents, contain bioactive compounds with notable antimicrobial properties.

In a comparative Analysis of the Antimicrobial Activity of *S. marianum* stems, leaves, and flowers, the petroleum extract of *S. marianum* flowers had the highest activity. In contrast, the leaf extract showed the weakest effect. While the Ethyl acetate extract of stems showed the highest inhibition, flowers and leaves exhibited similar antimicrobial effects. Additionally, the acetone extract of flowers had the highest inhibition against *S. aureus*, while the stems were most effective against *P. aeruginosa*. On the other hand, the Methanol extract of stems had the highest inhibition against *P. aeruginosa*, while flowers showed stronger activity against *S. aureus* and *C. albicans*. However, Water extracts had the weakest antimicrobial effects, with flower extracts being slightly more effective than those from stems and leaves. Iraqi et al. (2025) observed moderate antibacterial activity from leaves and stems of *S. marianum*, though seeds were most potent; notably, stems only inhibited *K. pneumoniae*.

In contrast, our multi-solvent leaf and flower extracts inhibited Gram-positive and Gram-negative strains with larger inhibition zones across five solvents. Similarly, Zhang et al. (2024) reported moderate antibacterial activity for stems and leaves, but seeds showed the highest potency. Our results extend this by demonstrating that flower extracts, especially using aqueous ethanol solvents, achieve broader and stronger inhibition zones across both Gram-positive and Gram-negative strains. Finally, our findings align with those of El-Sapagh et al. (2023), which concluded that aqueous–ethanolic seed extracts showed the strongest activity among solvents tested. However, our broader solvent gradient and plant part comparison allowed us to identify aqueous–ethanolic flower extracts as particularly potent against Gram-positive and Gram-negative pathogens, an insight not previously reported.

In the general conclusion of antimicrobial activity, methanol and acetone extracts showed the highest inhibition, especially against *S. aureus, P. aeruginosa,* and *C. albicans***.** Our findings indicate that methanol and acetone extracts did show an effect against all Gram-positive bacteria, Gram-negative bacteria, and fungi. In contrast, El-Sapagh et al. (2023) reported that the acetone extract had an antibacterial effect against MRSA but not against Gram-negative bacteria. Selim et al. (2025) reported that gram-negative bacteria, such as *E. coli*, are reported to have an outer lipopolysaccharide (LPS) layer on their surface that limits the entry of chemicals, which may explain their reduced sensitivity to antimicrobial agents. Ethyl acetate and petroleum extracts had moderate effects, while water extracts were the least effective.

The toxicity of flavonoids to microorganisms can be attributed to several mechanisms, including forming hydrogen bonds with proteins or enzymes in cell walls, extracting metal ions, inhibiting bacterial metabolism, and sequestering materials essential for bacterial growth (Wu et al. 2007). Plant extracts and various other phytochemical preparations rich in flavonoids have demonstrated antibacterial properties. Key antibacterial compounds include polyphenols such as tannins and flavonoids, including myricetin, quercetin, epigallocatechin, catechin, and luteolin (Kakran et al. 2012).

On the other hand, Flowers exhibited the highest antimicrobial activity across most solvent extracts, particularly against *S. aureus* and *B. subtilis*. The methanol extracts of *A. salviifolium* flowers have demonstrated a wide spectrum of antibacterial activity against Gram-positive and Gram-negative bacteria. Nanpazi, et al., (2020) reported that *S. marianum* flower extract exhibits strong antibacterial properties, especially against Gram-positive bacteria like *S. aureus*. Stems were most effective against *P. aeruginosa*, especially in methanol and acetone extracts. Leaves had moderate antimicrobial properties but were weaker compared to stems and flowers. Moreover*, P. aeruginosa* and *S. aureus* were the most susceptible strains, while *C. albicans* and *S. typhimurium* showed the lowest sensitivity to *S. marianum* extracts. Atef et al. (2019) reported that *P. aeruginosa* strains were susceptible to Ciprofloxacin.

**Fig. 8:**
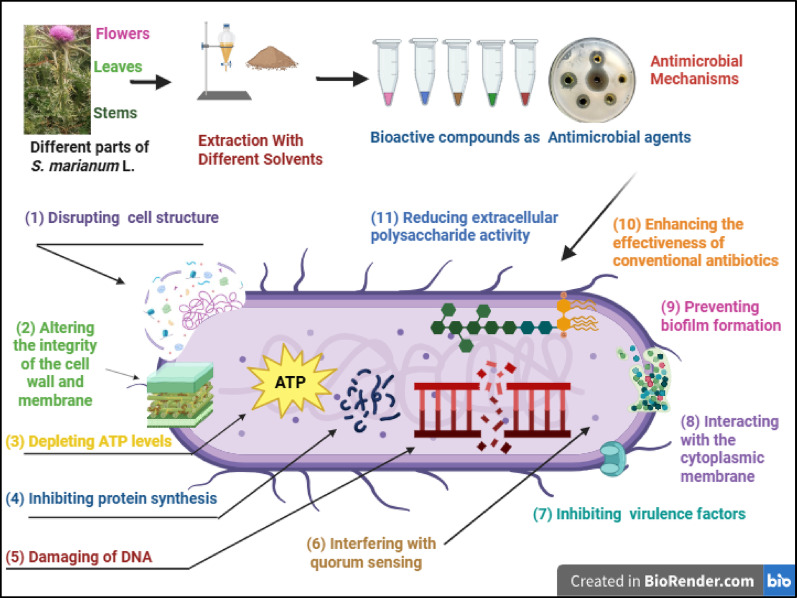
Hypothesized model of potential mechanisms of action of bioactive compounds of *S. arianum*

Medicinal plants such as *S. arianum* contain a wide range of bioactive compounds—including alkaloids, flavonoids, terpenoids, coumarins, tannins, antimicrobial peptides, and steroids—that can serve as alternatives or supplements to traditional antibiotics (AlSheikh et al. 2020; Fazly Bazzaz et al. 2021; Khameneh et al. 2021). These compounds exhibit antimicrobial effects through various hypothesized mechanisms (Fig. 8), such as (I) disrupting bacterial cell structure and increasing membrane permeability, leading to leakage of cellular contents; (II) altering the integrity of the cell wall and membrane; (III) depleting ATP levels; (IV) inhibiting protein synthesis; (V) causing damage within the cytoplasm, disturbing pH balance, and damaging DNA; and (VI) interfering with bacterial quorum sensing (Fatemi et al. 2020; Fazly Bazzaz et al. 2021; Gemeda et al. 2018; Khameneh et al. 2021). Additionally, polyphenols possess antibacterial properties that are effective against a wide range of bacterial species. Among these, flavanols, flavonols, and phenolic acids demonstrate the strongest activity by (I) inhibiting bacterial virulence factors such as enzymes and toxins, (II) interacting with the cytoplasmic membrane or lowering pH levels, (III) preventing biofilm formation, (IV) enhancing the effectiveness of conventional antibiotics through synergistic actions, and (V) reducing extracellular polysaccharide (EP) activity and functioning as EP inhibitors (EPIs) (Bazzaz et al. 2018; Miklasińska-Majdanik et al. 2018).

The current study's findings showed that various parts of *S. marianum* contain significant amounts of phenols, tannins, flavonoids, antimicrobials, and important proximate constituents. Significant economic value may be associated with the bioactive ingredients in S. marianum extracts and their potential as natural antioxidants. More research is required to determine which components have the strongest biological activities, including more thorough assays on the extraction, purification, and isolation of these compounds.

## Conclusions

The present study demonstrates that the stems, leaves, and flowers of *S. marianum* are rich sources of essential phytochemicals and phytonutrients utilized in ethnomedicine. The solvents used to extract *S. marianum* contain significant amounts of phenols, tannins, and flavonoids, contributing to its free radical scavenging activity. Methanol stands out as the most effective solvent compared to the others. It is capable of dissolving both hydrophilic and lipophilic compounds, is miscible with water, is less volatile, and serves as a highly efficient bioassay extractant. The study proves that different parts of *S. marianum* are rich in carbohydrates, proteins, and lipids, which are energy sources and have good medicinal potential. Moreover, *S. marianum* has health-beneficial nutritional properties, and its addition can improve disease resistance. For commercial reasons, more research is needed on identifying and purifying biochemical compounds. Methanol and acetone extracts demonstrated the strongest antimicrobial activity, particularly against *S. aureus*, *P. aeruginosa*, and *C. albicans*. Ethyl acetate and petroleum extracts showed moderate effects, whereas water extracts were the least effective. Among the different plant parts, flowers showed the strongest antimicrobial activity in most solvent extracts, especially against *S. aureus* and *B. subtilis*. Stems were particularly effective against *P. aeruginosa*, notably in methanol and acetone extracts. Leaves exhibited moderate antimicrobial activity but were less effective than stems and flowers. *P. aeruginosa* and *S. aureus* were the most sensitive strains, while *C. albicans* and *S. typhimurium* showed the least susceptibility to *S. marianum* extracts. Further research will focus on isolating and identifying active compounds from methanol and acetone extracts, exploring potential synergistic effects with antibiotics to improve activity against resistant strains, and assessing cytotoxicity and pharmacological potential before any clinical application.

## Supplementary Information

Below is the link to the electronic supplementary material.

Supplementary Material 1

## Data Availability

The data supporting the results of this study can be obtained from the corresponding author upon reasonable request.
