# Supplementary material for: Phytochemical analysis and antimicrobial activity of Silybum marianum L. via multi-solvent extraction
Source: AMB Express. 2025 Aug 20;15:122. doi: 10.1186/s13568-025-01925-2 (PMC12367635; doi:10.1186/s13568-025-01925-2)
Supplement: Supplementary file 1 — Supplementary Material 1 [file 13568_2025_1925_MOESM1_ESM.docx]

**Table 1S.** Preliminary qualitative phytochemical composition of dried stems, leaves, and flowers of *S. marianum*

| **Test** | **Stems** | **Leaves** | **Flowers** |
| --- | --- | --- | --- |
| Flavonoids | **++** | **++** | **+++** |
| Tannins | **+** | **++** | **+** |
| Alkaloids | **+** | **+** | **+** |
| Steroids | **+** | **+** | **++** |
| Saponins | **-** | **-** | **-** |
| Phenols | **+** | **++** | **+** |
| Anthraquinones | **+** | **+** | **+** |
| Glycosides | **+** | **++** | **++** |
| Terpenoids | **+** | **+** | **++** |
| Quinones | **+** | **+** | **+** |
| Cardiac glycosides | **++** | **+** | **+** |
| Anthocyanins | **-** | **-** | **-** |
| Coumarins | **-** | **+** | **++** |

+++: strongly, ++ moderately, + weak, - completely absent.

**Table 2S.** Summarizing of highest and minimum levels of secondary metabolites, associated *S. marianum* plant parts, and the most and least effective extraction solvents

| **Secondary metabolites** | **Values** | **Solvent** | **Part** |
| --- | --- | --- | --- |
| **Total phenolic content** |  |  |  |
| Highest value | 183.12±11.02 | Methanol | Flower |
| Lowest value | 5.45±1.32 | Water | Stem |
| **Total flavonoid content** |  |  |  |
| Highest value | 187.43±15.91 | Methanol | Flower |
| Lowest value | 9.60±1.5 | Water | Stem |
| **Total tannin content** |  |  |  |
| Highest value | 94.40±16.04 | Methanol | Flower |
| Lowest value | 3.27±1.53 | Water | Stem |
